# The role of bone flap explantation in the recurrence of surgical site infection after elective craniotomy: A multicenter propensity-matched cohort study

**DOI:** 10.1007/s10143-026-04291-0

**Published:** 2026-06-01

**Authors:** Kristin Lucia, Tim Schwarzmann, Malte Ottenhausen, Josephine Pollok, Vincent Prinz, Martin Barth, Jochen Tüttenberg, Florian Ringel, Marcus Czabanka

**Affiliations:** 1 Department of Neurosurgery, University Hospital Frankfurt am Main, Frankfurt, Germany; 2 Department of Neurosurgery, University Hospital of the Johannes Gutenberg University, Mainz, Germany; 3 Department of Neurosurgery, University of Heidelberg, Heidelberg, Germany; 4 Department of Neurosurgery, LMU University Hospital, Munich, Germany; 5 Department of Neurosurgery, Hospital of Frankfurt Höchst, Frankfurt, Germany; 6 Department of Neurosurgery, Hospital of Idar Oberstein, Idar-Oberstein, Germany

**Keywords:** Surgical site infection, Bone flap, Explantation, Craniotomy, Management

## Abstract

Surgical site infection (SSI) after craniotomy is a serious complication, and the optimal surgical strategy during revision remains controversial. This multicenter, propensity-matched cohort study compared outcomes between bone flap explantation and reimplantationin revision surgery and identified predictors of reinfection. A total of 160 patients undergoing revision surgery for SSI after elective craniotomy were included; 80 underwent bone flap reimplantation and 80 explantation. Patients were matched for age and FRAIL score. Clinical, radiological, microbiological, and surgical variables were analyzed, and predictors of recurrent SSI were assessed using Cox regression analysis. Recurrent SSI occurred significantly less often after bone flap explantation than reimplantation (18% vs. 49%, p = 0.001). Despite signs of more extensive infection in the explantation group, postoperative hospitalization was shorter (7 vs. 12 days, p = 0.051). Factors associated with reduced recurrence included infection limited to subcutaneous tissue, perioperative antibiotic administration, and shorter post-revision hospital stay. This study offers multicenter evidence that bone flap explantation is associated with substantially lower reinfection rates compared with immediate reimplantation which ultimately can improve outcomes and reduce healthcare burden.

**Supplementary Information:**

The online version contains supplementary material available at 10.1007/s10143-026-04291-0.

## Introduction

Postoperative surgical site infection (SSI) following craniotomy presents a significant challenge in the care of neurosurgical patients resulting in increased patient morbidity, prolonged treatment and economic burden for the health care system [1–3]. Rates of SSI following craniotomy have been described as ranging from 0.7 - 4.1% [4–10]. Most commonly, management of SSI entails surgical debridement followed by antibiotic therapy [11]. Debridement may be followed either by bone flap reimplantation or by bone flap removal (craniectomy), with later cranioplasty using alloplastic material or stored autologous bone. Despite the frequency of this clinical scenario, no evidence-based guidelines provide direction on whether immediate reimplantation or explantation reduces recurrence risk.

This study aimed to address this knowledge gap by performing a multicenter, propensity-matched comparison of bone flap reimplantation versus explantation during surgical revision for SSI after elective craniotomy. Additionally, independent predictors of SSI recurrence were evaluated to guide individualized surgical decision-making.

## Methods

We performed a retrospective review of patients who underwent elective cranial surgery with craniotomy at one University hospital from January 1, 2006, to December 31, 2020, and at a further University hospital and two tertiary care hospitals from January 1, 2018, to December 31, 2020. Inclusion criteria were elective craniotomy performed during the study period with at least one revision procedure indicated for wound-healing disorder at the craniotomy site, and age between 0 and 95 years. Exclusion criteria were emergency craniotomies, preexisting open skin lesions at the planned surgical site, initial burr hole trepanation only, age over 95 years; and the use of bone cement during the index procedure. Furthermore, patients without documented postoperative wound follow-up were excluded from analysis as lost to follow-up. Demographic data included age and sex. Relevant comorbidities were documented, focusing on diabetes mellitus, smoking history (including pack-years), and immunosuppressive conditions or therapies and FRAIL scores. Surgical indications were classified as tumor, vascular pathology, epilepsy surgery, or other. Operative parameters included date, duration (incision to closure), timing (regular hours vs. on-call), and surgeon experience (resident vs. attending), and intraoperative findings from revision surgery as recorded in surgical notes. All patients received a preoperative single-shot antibiotic which in general was a cephalosporine and was adapted in case of allergies. Wound-healing disorders were characterized by clinical signs (erythema, swelling, pain, dehiscence, necrosis, secretion), imaging findings indicating depth and localization of infection, and inflammatory markers (CRP, leukocyte count). Revision surgery was classified as debridement with or without permanent bone flap removal (craniectomy). While the decision to perform craniectomy was ultimately left to the discretion of the individual surgeon, signs of intracerebral, subdural and in most cases also epidural infection, generally implicated explantation of the bone flap. Microbiological cultures from intraoperative swabs and subsequent antibiotic therapies were recorded. Postoperative follow-up assessments documented wound status, including recurrence of wound complications, imaging, laboratory data, and additional revision surgeries. For patients undergoing craniectomy, subsequent bone flap reimplantation and timing were also noted. Informed consent was obtained from all individual participants included in the study. This study was performed in line with the principles of the Declaration of Helsinki. Approval was granted by the Ethics Committee of Medical Faculty of the Goethe University (June 22, 2022/Nr. 2022 − 824). No funding was received for conducting this study.

All analyses were performed using SPSS version 29 (IBM Corp., Armonk, NY).

Propensity score matching (PSM) was performed 1:1 on age and FRAIL score using nearest-neighbor matching without replacement and a caliper width of 0.2 SD of the logit of the propensity score. Balance was assessed using standardized mean differences (< 0.1 considered adequate). After matching, comparisons between groups used Mann–Whitney U tests (continuous variables) and chi-square or Fisher’s exact tests (categorical variables). Univariate Cox regression assessed predictors of recurrent SSI; hazard ratios (HRs) with 95% confidence intervals (CIs) were reported. p-values < 0.05 were considered statistically significant.

## Results

### Patient characteristics

A total of 160 patients (80 explantation, 80 reimplantation) were included. After PSM, both groups had a mean age of 52 years, and FRAIL score distribution was identical, with 40 patients per group having a score of 0. Gender distribution was equal (48% male, *p* = 1.00) (Table 1). Comorbidities—including corticosteroid use, immunosuppression, diabetes, chemotherapy, and smoking—did not differ significantly between groups. Corticosteroid use was the most common comorbidity (28% vs. 31%, *p* = 0.729). Surgical indication was most often tumor resection (89% vs. 86%, *p* = 0.818). Index procedure durations were comparable (280 vs. 284 min), as were the proportions performed by attending neurosurgeons (*p* = 0.356) (Table 1). 

**Table 1:** Demographic characteristics of patient cohort

	Bone flap explanted	Bone flap reimplanted	*p*
Total Patients *n* (% of total)	80 (50%)	80 (50%)	*n*.a
Age in years Mean (standard deviation)	52 (13)	52 (14)	0.837
Gender n (% of total) Male Female	38 (48%) 42 (52%)	38 (48%) 42 (52%)	1.00
FRAIL Score 0 1 2 3 4	40 25 9 5 1	40 25 14 1 0	0.294
Comorbidities Oral Corticosteroids Immunosuppression Diabetes mellitus Radiation therapy Chemotherapy ongoing Smoking*	22 11 7 15 13 14	25 13 7 20 13 12	0.729 0.413 0.610 0.339 0.585 0.105

*Information missing for 43 patients undergoing bone flap explantation and 32 Patients undergoing bone flap reimplantation. Distribution of age and frailty scores following PMSA

The most common first surgical procedure requiring elective craniotomy among both cohorts was for an intracranial tumor (89% in bone flap explanted patients and 86% in bone flap reimplanted patients) (Table 2). Overall, there was no statistically significant difference in the distribution of surgical procedures performed in the first surgery among both cohorts (*p* = 0.818). Among patients undergoing bone flap explantation, the first surgical procedures lasted an average of 280 min and in the bone flap reimplantation group 284 min (*p* = 0.154). In the bone flap explantation group, prophylactic antibiotic administration was performed in 39 patients (86%). Among patients undergoing bone flap reimplantation, perioperative antibiotic administration was performed in 15 patients (83%) (*p* = 0.707). In both groups most initial surgical procedures were performed by a board-certified neurosurgeon (70 patients in the bone flap reimplantation group and 66 in the bone flap explantation group) (*p* = 0.356). In all other cases surgery was performed by a neurosurgical resident. The average length of inpatient hospitalization was 19 days (SD 79) in the bone flap explantation group and 25 (SD 111) days in the bone flap reimplantation group (*p* = 0.832). Additional information regarding type and size of craniotomy as well as the use of single-shot preoperative antibiotics can be found in supplementary tables S1-S3. 

**Table 2:** First surgical procedure/craniotomy

	Bone flap explanted	Bone flap reimplanted	*p*
Total Patients *n* (% of total)	80 (50%)	80 (50%)	
First Surgical Procedure Tumor Vascular Epilepsy Other	71 (89%) 4 (5%) 1 (1%) 4 (5%)	69 (86%) 6 (8%) 0 (1%) 5 (5%)	0.818
Length of first surgical procedure Mean (Standard Deviation) in minutes	280 (215)	284 (216)	0.154
Level of training of surgeon performing first surgical procedure Neurosurgical Resident Board Certified Neurosurgeon	10 70	14 66	0.356
Inpatient stay for first surgical procedure Mean (Standard Deviation) in days	19 (79)	25 (111)	0.832

Details of the first surgical procedure performed (initial craniotomy) prior to SSI

### Characteristics of initial SSI following craniotomy

Time from index craniotomy to SSI revision was similar across groups (176 vs. 151 days, *p* = 0.712). Large standard deviations within these groups can be accounted for due to four cases (two per group), in which SSI occurred between one and three years following index craniotomy. Revision surgeries were performed more frequently outside regular hours in the explantation cohort (53 vs. 29 cases, *p* = 0.001) (Table 3). Surgical revision of SSI was performed by board certified Neurosurgeons in 44 cases and by neurosurgical residents in 36 cases in both patient groups. Revision surgeries were performed more frequently outside regular hours in the explantation cohort (53 vs. 29 cases, *p* = 0.001) (Table 3). The length of surgical procedures did not differ significantly between groups (64 min for bone flap explantation and 70 min for bone flap reimplantation; *p* = 0.146). Patients undergoing bone flap explantation had shorter inpatient stays than those undergoing bone flap reimplantation (7 versus 12 days; *p* = 0.051). Increased standard deviations in this category were not related to issues of wound healing. Records of clinical presentation of SSI revealed that CSF fistulas were significantly more common in the reimplantation group (13 vs. 2 cases, *p* = 0.022). Abnormal systemic inflammatory markers were more frequent in the explantation cohort (63 vs. 53 cases, *p* = 0.037). Radiological assessment of the deepest extent of infection showed the most common finding in both groups was involvement of the epidural space (29% of bone flap explantations and 35% of bone flap reimplantations). CSF fistulas were noted radiologically more often in the reimplantation cohort (11% vs. 0%, *p* = 0.05). Intracerebral involvement was more common in the explantation group (26% vs. 13%, *p* = 0.05).

**Table 3:** Characteristics of initial SSI following elective craniotomy

	Bone flap explanted	Bone flap reimplanted	*p*
Total Patients *n* (% of total)	80 (50%)	80 (50%)	*n*.a
Time since first surgery Mean (standard deviation) in days*	176 (950)	151 (856)	0.712
Level of training of surgeon performing surgical procedure Neurosurgical Resident Board certified Neurosurgeon	36 44	36 44	0.434
Surgery performed during regular operating hours (07:00–16:00) Yes No	27 53	51 29	***0.001***
Length of surgery Mean (Standard Deviation) in Minutes	64 (81)	70 (110)	0.146
Inpatient stay Mean (Standard Deviation) in Days	7 (7)	12 (7)	0.051
Clinical wound assessment findings N (% of total) Dehiscence Necrosis Secretion Swelling Erythema Hematoma CSF fistula	34 (43%) 2 (2%) 54 (68%) 17 (21%) 15 (19%) 0 (0%) 2 (2%)	24 (30%) 2 (2%) 48 (60%) 18 (22%) 8 (10%) 1 (1%) 13 (1%)	0.139 0.690 0.411 1.000 0.175 1.000 ***0.022***
Abnormal systemic infection parameters	63 (79%)	53 (66%)	***0.037***

Clinical details of first SSI following craniotomy in both cohorts

**Fig. 1:**
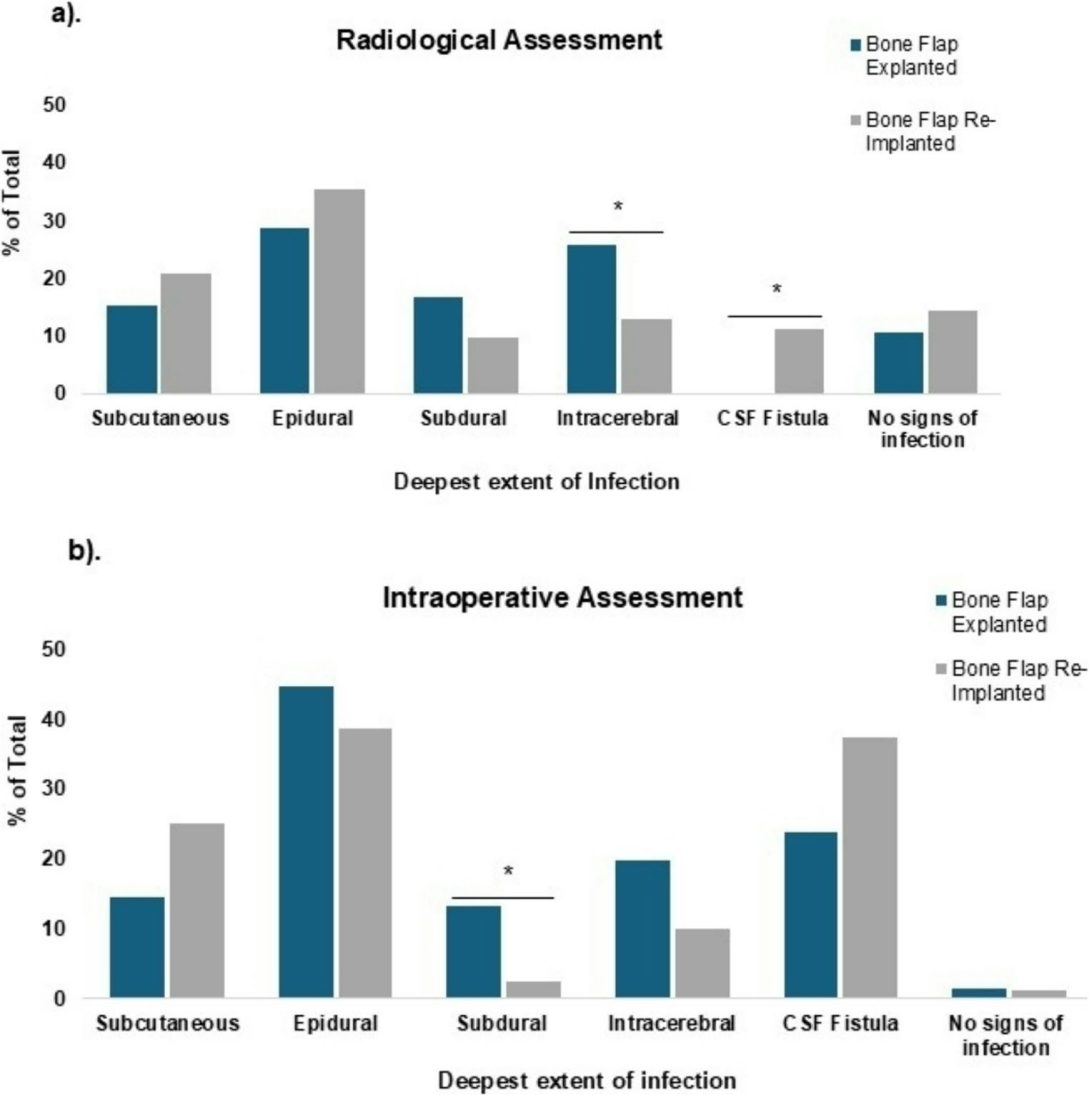
Preoperative radiological assessment of patients undergoing revision surgery for a postoperative wound healing disorder was performed to determine the deepest extent of possible infection. Extent of infection according to intraoperative assessment by the surgical team was also recorded. Results are shown for groups of patients undergoing explantation versus reimplantation of bone flaps are shown with p < 0.05 considered statistically significant.

Absence of radiological infection signs occurred in 11% and 15% of patients, respectively (Fig. 1). In addition to the radiological description of the extent of infection, surgical reports were also used to document the intraoperative assessment of the neurosurgeon regarding possible differences in the extent of infection in situ. Overall, the general pattern of intraoperative findings closely resembled those of radiological assessments. While epidural extent of infection was also the most common finding intraoperatively, the actual rates were higher than reported in radiological reports (45% in bone flap explantation and 39% in bone flap reimplantations). CSF fistulas were also more commonly reported intraoperatively than on radiological reports (24% of bone flap explantations and 38% of bone flap reimplantations). In contrast to radiological reports, subdural extent of infection was observed intraoperatively at higher rates among bone flap explanted patients (13%) versus bone flap reimplanted patients (3%) (*p* = 0.032). Furthermore, surgical assessment revealed that only 1% of patients in both groups showed no signs of infection, whereas radiological findings estimated that 11% and 15% of patients in the bone flap explanted and reimplanted groups had no sign of infection.

### Pathogen spectrum in first SSI

**Fig. 2:**
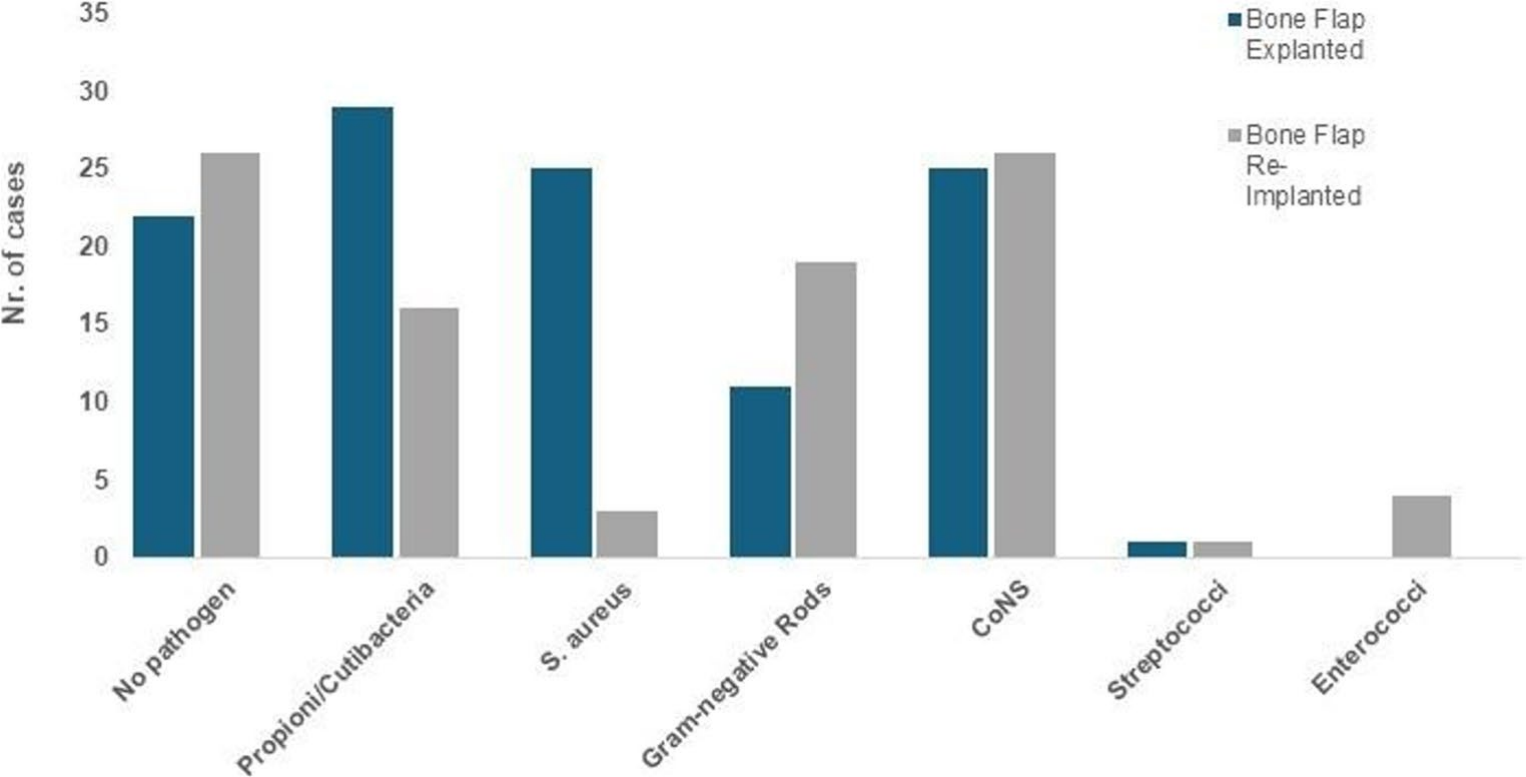
Results of microbiological results from intraoperative sampling at the deepest location of infection. Results are presented as absolute counts in patients with and without reimplantation of bone flap. CoNS = Coagulase negative staphylococci.

Intraoperative sampling results for microbiological analysis revealed no significant differences between groups of bone flap explanted and reimplanted patients (Fig. 2). Overall, no pathogens could be identified in 20 patients with bone flap explantation and 26 patients undergoing bone flap reimplantation. The most commonly found pathogens were Propioni- or cutibacteria acnes (29 cases) followed by Staphylococcus aureus (25 cases) in bone flap explanted patients. Among patients undergoing bone flap reimplantation coagulase negative staphylococci were most frequently observed in 25 cases, followed by gram negative rods (19 cases) (Fig. 2). Detailed information regarding postoperative antibiotic therapy following surgical revision can be found in supplementary table S4.

### Frequency of repeat SSI following initial surgical revision

Evaluation of the overall recurrence rate of SSI following initial surgical revision procedures revealed that patients undergoing bone flap reimplantation displayed significantly lower rates of recurrent infection compared to those in which the bone flap was reimplanted following surgical debridement (18% vs. 49%, *p* = 0.001) (Table 4).

**Table 4:** Frequency of repeat SSI following initial surgical revision

	Bone flap explanted	Bone flap re-implanted	p
Recurrent Wound Healing disorder N (% of total population)	15 (18%)	39 (49%)	***0.001***

McNemar test of significance between patients undergoing bone flap reimplantation versus those with bone flap explantation. *P* < 0.05 is considered significant

### Predictors from first revision surgery for the recurrence of SSI

Univariate Cox Regression analysis was performed on various parameters of primarily SSI regarding their possible utility as a predictor for the recurrence of a further SSI. This analysis was performed on all 160 patients included in the study (Table 5). Although the rate of recurrent SSI infection was significantly higher among patients undergoing bone flap reimplantation, bone flap management itself was not identified as an independent predictor (HR 1.636; CI 0.901–2.970, *p* = 0.106). Among preoperative factors, radiological evidence of infection extending no deeper than the subcutaneous space was associated with a significantly reduced rate of recurrent SSI (HR 0.488; CI 0.258–0.992; *p* = 0.027). Analysis of surgical and intraoperative parameters revealed that the administration of perioperative antibiotics also led to a significant reduction in the rate of recurrent SSI (HR 0.344; CI 0.179–0.660; *p* = 0.001). No significant association between particular microbiological findings were associated with the recurrence of SSI. Finally, a shorter inpatient stay following primary surgical debridement (as measured by the median of all values) was significantly associated with a reduction in the rate of recurrence of SSI (HR 0.454; CI = 0.256–0.808; *p* = 0.007).

**Table 5:** Predictive factors from first revision surgery for the recurrence of SSI

Event	Hazard Ratio	CI (95%)	*P*
Preoperative Assessment Abnormal systemic infection parameters Subcutaneous infection on imaging Epidural infec tion on imaging Subdural infection on imaging Intracerebral infection on imaging CSF Fistula on imaging	1.014 0.488 0.786 1.750 1.380 0.980	0.516–5.175 0.258–0.922 0.429–1.438 0.625-4.900 0.543–3.510 0.349–2.755	0.967 ***0.027*** 0.435 0.287 0.499 0.970
Surgical Parameters Explantation of Bone Flap Surgery performed by board certified Neurosurgeon Surgery time over 58 Min* Use of subcutaneous wound drain Administration of perioperative Antibiotics	1.636 1.224 0.832 1.222 0.344	0.901–2.970 0.710–2.111 0.479–1.445 0.710–2.103 0.179–0.660	0.106 0.514 0.468 ***0.001***
Microbiological Findings Coagulase negative Staph. S. aureus (MSSA+MRSA) Propionibacterium/cutibacterium acnes Streptococci Enterococci Gram negative rods	1.123 1.000 1.270 20.730 20.452 0.820	0.502–2.502 0.551–1.815 0.613–2.630 0.001–485,182 0.000-382119 0.367–1.832	0.519 0.999 0.519 0.555 0.682 0.629
Postoperative parameters Length of inpatient stay < 11 days*	0.454	0.256–0.808	***0.007***

Univariate Cox Regression analysis was performed using the listed variables as a predictor of the recurrence of a wound-healing disorder which has already been surgically treated. All variables are those which were recorded at the time of the first revision surgery. * Values set based on the median of all cases. *P* < 0.05 is considered significant

### Characteristics of recurrent SSI among patients undergoing secondary cranioplasty

Among 80 patients undergoing primary bone flap explantation within the first revision surgery for SSI, follow-up data regarding secondary cranioplasty was available for 76 patients (95%). Among these patients, 44 cases underwent secondary cranioplasty (58%) and 32 did not (42%). All cases of secondary cranioplasty were using an allogenic implant. The average time between bone flap explantation and reimplantation was 288 days. Further data on wound healing was available for 39 patients following completion of secondary cranioplasty. Among these patients, seven developed a further SSI (18%). Wound secretion was the most common clinical symptom among recurrent SSI in this group (57%). Six of these patients underwent further surgical revision, with one case being managed conservatively.

## Discussion

This multicenter propensity-matched analysis demonstrates that bone flap explantation during revision surgery for post-craniotomy SSI yields significantly lower reinfection rates compared with immediate reimplantation. Definitive treatment may therefore be achieved sooner in those undergoing secondary cranioplasty following debridement procedures.

Previous literature presents mixed findings regarding optimal bone flap management. A recent meta-analysis reported lower treatment failure with immediate cranioplasty (10.4%) compared with delayed (16.1%), yet most included studies were small and heterogeneous [12]. Only two of the fifteen studies included in this meta-analysis directly intended to compare the role of immediate versus delayed implantation of bone flap in SSI surgery. The first found significantly lower rates of recurrent infection when delayed (4.5%) versus immediate (26%) cranioplasty was performed [13]. The second study found no differences in the rate of recurrent SSI upon different management strategies for bone flap reimplantation (immediate reimplantation after washing, craniectomy or titanium cranioplasty at the time of surgical debridement). Among patients undergoing reimplantation of the bone flap during debridement surgery, there was a significantly higher rate of recurrent infection when purulence was observed, pointing towards the role of the bone flap as microbial reservoir [14].

As could be shown in a further case series of 11 patients who underwent surgical debridement for SSI following tumor resection, direct reimplantation of the bone flap resulted in recurrent SSI in only one patient (10%) [15]. While significantly lower than the reinfection rates found in patients undergoing primary cranioplasty in this study, the sample size of 11 patients strongly limits further generalization in clinical practice.

Our findings that microorganisms were isolated at similar frequencies across groups supports the hypothesis that reinfection risk is not driven solely by initial pathogen burden but may relate to persistent contamination of the autologous bone flap.

Abnormal systemic infection parameters were also more often found among patients undergoing bone flap explantation than bone flap reimplantation (63 versus 53 cases (0.037). Taken together, our findings support the theory that retention of a potentially infected bone flap following surgical debridement may represent a bacterial reservoir which in turn promotes recurrent infection. Explantation of this reservoir can therefore lower reinfection rates.

Furthermore, we hypothesized that procedures conducted outside of regular operating hours may reflect a higher degree of clinical urgency, potentially due to more severe clinical or radiological manifestations of infection. In our analysis, a greater proportion of patients in the bone flap explantation cohort underwent surgery outside of scheduled operating hours (51 cases, 64%) compared to the bone flap reimplantation cohort (29 cases, 36%), a difference that was statistically significant (*p* = 0.001). The significantly higher frequency of off-hours surgery in the explantation group suggests more severe or urgent presentations. Despite this, outcomes favored explantation, indicating that bone flap removal confers therapeutic benefit even in advanced infections.

Our data indicated that patients who underwent bone flap explantation had shorter inpatient stays compared to those who underwent bone flap reimplantation (7 vs. 12 days; *p* = 0.051). This finding suggests the possibility that patients receiving explantation may experience more rapid clinical improvement, facilitating earlier discharge. However, this hypothesis warrants further investigation in future studies.

Review of clinical records revealed that cerebrospinal fluid (CSF) fistulas were significantly more frequent among patients who underwent subsequent bone flap reimplantation compared to those who underwent bone flap explantation (13 vs. 2 cases; *p* = 0.022). The presence of a CSF fistula in these cases may have influenced the decision to reimplant the bone flap. While the overall findings of this study support bone flap removal as a more effective strategy for managing surgical site infections (SSI), the presence of a concurrent CSF fistula may define a distinct clinical subgroup that warrants special consideration in surgical decision-making.

Bone flap explantation during surgical revision for post-craniotomy SSI is associated with significantly lower reinfection rates compared with immediate reimplantation, even in patients exhibiting more severe infection markers. Perioperative antibiotics, subcutaneous-only infection, and shorter hospitalization further reduce recurrence risk. These findings support explantation as a preferred strategy for managing SSIs after elective craniotomy and highlight opportunities to standardize care pathways to improve outcomes and reduce healthcare burden.

Limitations of the current study include its retrospective nature as well as the non-standardized surgical protocols across centers for procedures included in the analysis.

## Supplementary Information

Below is the link to the electronic supplementary material.

Supplementary Material 1 (DOCX 18.5 KB)

## Data Availability

No datasets were generated or analysed during the current study.

## Declarations

No funds, grants, or other support was received.
