# Supplementary material for: The role of bone flap explantation in the recurrence of surgical site infection after elective craniotomy: A multicenter propensity-matched cohort study
Source: Neurosurg Rev. 2026 Jun 1;49(1):428. doi: 10.1007/s10143-026-04291-0 (PMC13226313; doi:10.1007/s10143-026-04291-0)
Supplement: Supplementary file 1 — Supplementary Material 1 (DOCX 18.5 KB) [file 10143_2026_4291_MOESM1_ESM.docx]

**Table S1: Craniotomy performed for initial surgical procedures**

| **Craniotomy** | **Total**  **N=160** | **Bone Flap Explanted**  **N=80** | **Bone flap re-implanted**  **N=80** | **p** |
| --- | --- | --- | --- | --- |
| Convexity | 117 (73,13 %) | 56 (70,00 %) | 61 (76,25 %) | 0,373 |
| Pterional/Supra-orbital | 30 (18,75 %) | 16 (20,00 %) | 14 (17,50 %) | 0,685 |
| Retrosigmoidal | 5 (3,13 %) | 4 (5,00 %) | 1 (1,25 %) | 0,367 |
| Suboccipital/posterior fossa | 8 (5,00 %) | 4 (5,00 %) | 4 (5,00 %) | 1,000 |

**Table S2: Craniotomy size from initial surgical procedures**

| **Craniotomy Size** | **Total**  **N=130** | **Bone Flap Explanted**  **N=74** | **Bone Flap Re-implanted**  **N=56** | **p** |
| --- | --- | --- | --- | --- |
| Mean | 28,53 | 29,27 | 27,55 | 0,712 |
| Standard Deviation | 19,75 | 20,53 | 18,82 |  |
| Minimum | 2,87 | 3,42 | 2,87 |  |
| Maximum | 100,92 | 100,92 | 71,37 |  |

**Table S3: Preoperative single-shot antibiotic administration before initial surgical procedure**

| **Antibiotic** | **Total**  **N=49** | **Bone Flap Explanted**  **N=33** | **Bone Flap Re-implanted**  **N=16** | **p** |
| --- | --- | --- | --- | --- |
| Cephalosporine | 32 (65,31 %) | 24 (72,73 %) | 8 (50,00 %) | 0,086 |
| Aminoglycoside | 8 (16,33 %) | 1 (3,03 %) | 7 (43,75 %) | **<0,001** |
| Clindamycin | 2 (4,08 %) | 1 (3,03 %) | 1 (6,25 %) | 1,000 |
| Fosfomycin | 1 (2,04 %) | 1 (3,03 %) | 0 (0,00 %) | 1,000 |
| Vancomycin | 1 (2,04 %) | 0 (0,00 %) | 1 (6,25 %) | 0,325 |

**Table S4: Antibiotic therapy following first revision surgery for SSI**

| **Antibiotic** | **Total**  **N=153 (101+52)** | **Bone flap explanted**  **N=78** | **Bone flap re-implanted**  **N=75** | **p** |
| --- | --- | --- | --- | --- |
| Cephalosporine | 15 (9,80 %) | 13 (16,67 %) | 2 (2,67 %) | **0,005** |
| Aminoglykoside | 1 (0,65 %) | 1 (1,28 %) | 0 (0,00 %) | 1,000 |
| Clindamycin | 22 (14,38 %) | 11 (14,10 %) | 11 (14,67 %) | 0,921 |
| Fosfomycin | 0 (0,00 %) | 0 (0,00 %) | 0 (0,00 %) | const. |
| Penicilline und Aminopenicilline | 3 (1,96 %) | 3 (3,85 %) | 0 (0,00 %) | 0,245 |
| Vancomycin | 54 (35,29 %) | 27 (34,62 %) | 27 (36,00 %) | 0,858 |
| Carbapeneme | 56 (36,60 %) | 28 (35,90 %) | 28 (37,33 %) | 0,854 |
| Rifampicin | 2 (1,31 %) | 2 (2,56 %) | 0 (0,00 %) | 0,497 |
| Metronidazol | 0 (0,00 %) | 0 (0,00 %) | 0 (0,00 %) | const. |
| Fluorchinolone | 1 (0,65 %) | 0 (0,00 %) | 1 (1,33 %) | 0,490 |
| Linezolid | 4 (2,61 %) | 4 (5,13 %) | 0 (0,00 %) | 0,120 |
| Cotrimoxazol | 2 (1,31 %) | 2 (2,56 %) | 0 (0,00 %) | 0,497 |
| Andere Antibiotika | 0 (0,00 %) | 0 (0,00 %) | 0 (0,00 %) | const. |
